# Does conventional specimen radiography after neoadjuvant chemotherapy of breast cancer help to reduce the rate of second surgeries?

**DOI:** 10.1007/s10549-021-06466-3

**Published:** 2021-12-08

**Authors:** Benedikt Schaefgen, Annika Funk, H.-P. Sinn, Thomas Bruckner, Christina Gomez, Aba Harcos, Anne Stieber, Annabelle Haller, Juliane Nees, Riku Togawa, André Pfob, André Hennigs, Johanna Hederer, Fabian Riedel, Sarah Fastner, Christof Sohn, Jörg Heil, Michael Golatta

**Affiliations:** 1 Department of Gynecology and Obstetrics, University of Heidelberg, Heidelberg, Germany; 2 Department of Radiology, University of Heidelberg, Heidelberg, Germany; 3 Department of Pathology, University of Heidelberg, Heidelberg, Germany; 4 IMBI- Institute for Medical Biometry and Informatics University of Heidelberg, Heidelberg, Germany; 5 Universitätsfrauenklinik, INF 440, 69120 Heidelberg, Germany; 6 Pathologisches Institut, INF 224, 69120 Heidelberg, Germany; 7 IMBI, INF 130.3, 69120 Heidelberg, Germany; 8 Klinik Für Radiologie, INF 110, 69120 Heidelberg, Germany

**Keywords:** Breast cancer, Breast conserving therapy, Surgical margins, Intraoperative re-excision, Specimen radiography, Neoadjuvant chemotherapy

## Abstract

**Purpose:**

This is the first study to systematically evaluate the diagnostic accuracy of intraoperative specimen radiography on margin level and its potential to reduce second surgeries in patients treated with neoadjuvant chemotherapy.

**Methods:**

This retrospective study included 174 cases receiving breast conserving surgery (BCS) after neoadjuvant chemotherapy (NACT) of primary breast cancer. Conventional specimen radiography (CSR) was performed to assess potential margin infiltration and recommend an intraoperative re-excision of any radiologically positive margin. The histological workup of the specimen served as gold standard for the evaluation of the accuracy of CSR and the potential reduction of second surgeries by CSR-guided re-excisions.

**Results:**

1044 margins were assessed. Of 47 (4.5%) histopathological positive margins, CSR identified 9 correctly (true positive). 38 infiltrated margins were missed (false negative). This resulted in a sensitivity of 19.2%, a specificity of 89.2%, a positive predictive value (PPV) of 7.7%, and a negative predictive value (NPV) of 95.9%. The rate of secondary procedures was reduced from 23 to 16 with a number needed to treat (NNT) of CSR-guided intraoperative re-excisions of 25.

In the subgroup of patients with cCR, the prevalence of positive margins was 10/510 (2.0%), PPV was 1.9%, and the NNT was 85.

**Conclusion:**

Positive margins after NACT are rare and CSR has only a low sensitivity to detect them. Thus, the rate of secondary surgeries cannot be significantly reduced by recommending targeted re-excisions, especially in cases with cCR. In summary, CSR after NACT is inadequate for intraoperative margin assessment but remains useful to document removal of the biopsy site clip.

## Introduction

In the past decades, breast conserving surgery (BCS) has become the standard surgical approach for early breast cancer, and leads to equal [1, 2] or superior [3, 4] overall survival compared to mastectomy. Neoadjuvant chemotherapy (NACT) is the standard approach for high-risk cancer patients and can lead to a significant reduction of tumor mass. Often this allows a further reduction of the extent of breast surgeries, which contributes to an improvement of the esthetic outcome and patient satisfaction [5–7] as well as a higher quality of life [8, 9] and a reduced risk of postoperative complications. The surgeon should try to remove as little healthy tissue as possible while avoiding tumor-infiltrated (positive) resection margins, which are a risk factor for local recurrence [10]. Conventional specimen radiography (CSR) using mammography in two orthogonal orientations is used to assess the margin status and recommend intraoperative re-excision if necessary. Ideally, this leads to tumor free resection margins and can help to avoid a secondary re-excision. The proportion of patients who actually benefit from CSR depends on the prevalence of initially positive margins. Thanks to more frequent administration and more effective systemic treatment options, an increasing number of patients achieve a pathological complete response (pCR) after NACT [14]. Thus, the prevalence of initially positive margins is expected to be lower in patients after NACT and the use of CSR seems questionable in the postneoadjuvant setting [15].

## Materials and methods

This study was approved by the ethics committee of the University’s Medical Faculty under file number S-468/2016.

### Patient population

**Fig. 1:**
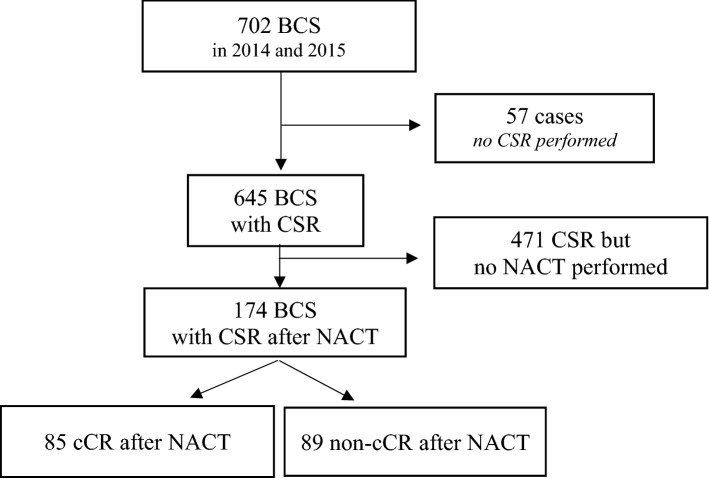
Flow diagram of patient population

Patients treated at the Breast Unit with BCS after NACT of invasive breast cancer between January 2014 and December 2015 were included consecutively in the analysis. Cases that did not receive CSR (*n* = 57), mostly for reasons of palpability, or did not receive NACT (*n* = 471) were excluded from further analysis. For subgroup analysis, patients´ response to NACT was classified as clinical complete response (cCR; defined as the absence of evidence of residual tumor in clinical examination, ultrasound, and mammography after NACT) and non-cCR (Fig. 1).

### Conventional specimen radiography and surgical procedure

Preoperative wire-localization using ultrasound or stereotactic guidance was performed and controlled by mammography.

The specimen orientation was marked by sutures of different lengths according to institutional standard operating procedures. CSR was performed in the breast unit using Mammomat Inspiration (Siemens AG, Erlangen, Germany) with 1.4 × direct magnification in two orthogonal views without compression. One of six physicians with more than ten years of experience in diagnostic mammography and CSR evaluated the position of the target lesion and its relation to the resection margins. Clinical history and previous images were available to the radiologist. If any of the margins appeared to be infiltrated, the radiologist advised the surgeon to perform an intraoperative re-excision of the same orientation.

**Fig. 2:**
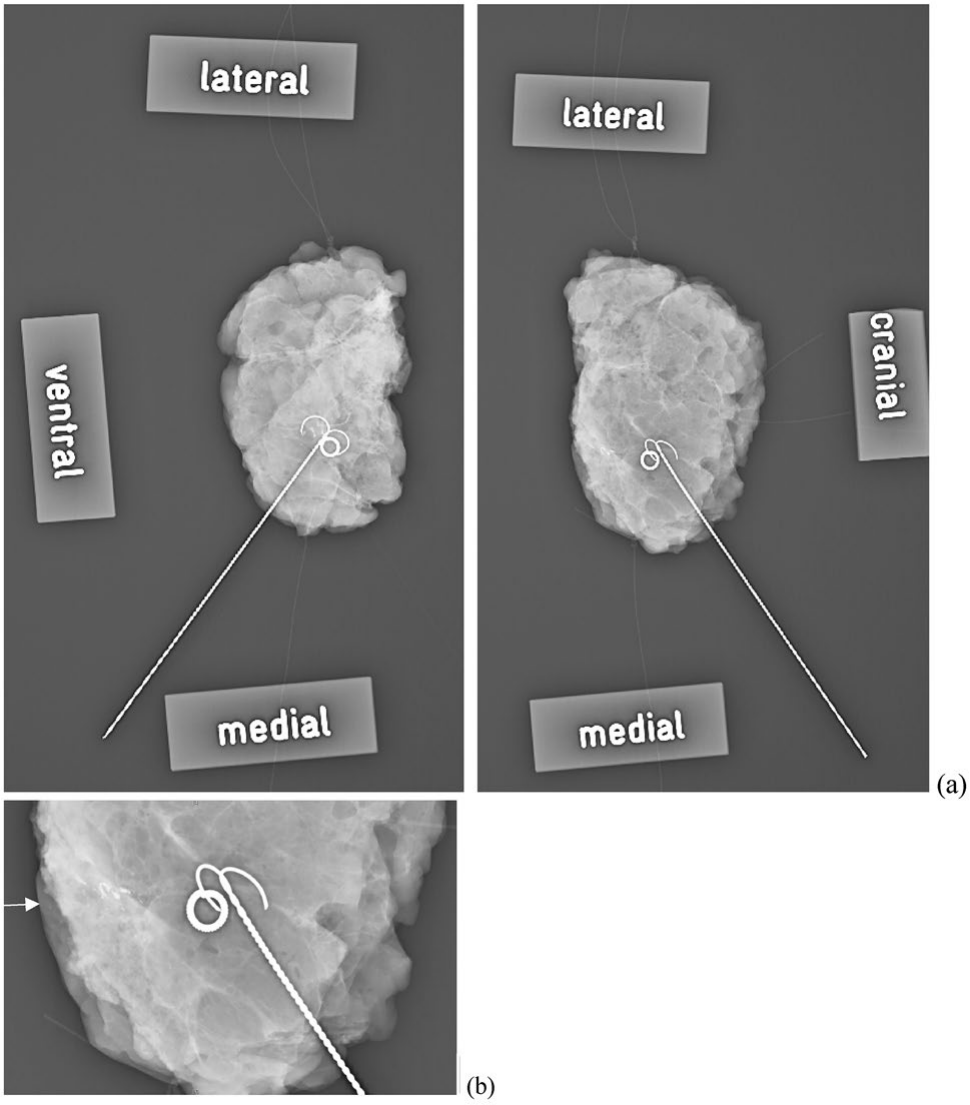
Example of a conventional two-view specimen radiograph of a cCR patient. Marking wire and clipmarker are visible in the former tumor bed (**a**). In the twofold magnification of CSR, residual microcalcifications with insufficient margin width in the dorsal direction are visible, so re-excision was recommended in this direction (**b**). In contrast, the pathological workup showed a pCR (false positive CSR). The arrow indicates residual microcalcifications reaching the caudal margin

The pathologic workup of the specimen and the re-excisions was the gold standard for the evaluation of the diagnostic accuracy of CSR. According to the national guideline at the time of data collection (2014–2015), a positive margin was defined as < 1 mm in invasive carcinoma and < 2 mm in ductal carcinoma in situ (DCIS) [16], defining the indication for re-excision. According to the guideline of 2017, a clear margin was defined as “no ink on tumor” [17] (Fig. 2).

### Statistical analysis

Descriptive analyses were performed using IBM SPSS Statistics Version 26, (Armong, NY, USA). Sensitivity and specificity of CSR was calculated along with 95% confidence intervals using SAS 9.4 WIN (Cary, NC, USA). One sided Chi-square-test was used to assess the level of significance of the differences in sensitivity and specificity among the subgroups. P-values are not adjusted for multiplicity and must be interpreted descriptively.

The primary endpoint was the NNT to avoid a second surgery by CSR-guided intraoperative resections.

## Results

174 patients received BCS after NACT and were included in this analysis (Table 1). For each primary resection specimen, six margins were assessed (1044 in total).

**Table 1:** Patient and tumor characteristics

Number of patients	(*n* = 174) (percentages in brackets)
Age	
Mean	51.4 (12.2)
Range	24 to 82
Ethnicity	not systematically assessed, mostly European
Cup size	
A	5 (2.9)
B	46 (26.4)
C	26 (14.9)
D	14 (8.0)
E	2 (1.1)
F	1 (0.6)
Unknown	80 (45.4)
Menopausal status	
Premenopausal	62 (35.6)
Menopausal	22 (12.6)
Postmenopausal	90 (51.7)
Breast density (ACR)	
A	11 (6.3)
B	82 (47.1)
C	61 (35.1)
D	20 (11.5)
Target structure for lesion marking	
Clip marker	158 (90.8)
Clip marker detected in CSR	154 (97.4)
Microcalcifications	16 (9.2)
Radiographic presentation of the tumor	
Only mass	126
Mass with microcalcifications	16
Only microcalcifications	2
MRI performed	
Before and after NACT	18 (10.3)
Only before NACT	68 (39.1)
Only after NACT	5 (2.8)
Remission status	
cCR	85 (48.9)
With pCR (%)	58 (68.2)
Non-cCR	89 (51.1)
With pCR (%)	25 (28.1)
Final T-stadium (ypT)	
0	82 (47.1)
is	11 (6.3)
1	62 (35.6)
– 1mic	1 (0.6)
– 1a	19 (10.9)
– 1b	14 (8.0)
– 1c	28 (16.1)
2	17 (9.8)
3	1 (0.6)
4	–
Median specimen weight	
Primary resection	38.0 g (range 5; 268)
Re-resection	7.6 g (range 1; 41)
Histologically Infiltrated margins by orientation	
Total	47 (4.5)
Medial	3 (0.3)
Lateral	3 (0.3)
Kranial	14 (1.3)
Kaudal	9 (0.9)
Ventral	4 (0.4)
Dorsal	14 (1.3)

*cCR* clinical complete response, *pCR* pathological complete response

85 Patients (48.9%) had a clinical complete response (cCR), whereas 89 (51.1%) had no cCR. In 82 cases (47.1%), NACT resulted in a pCR (ypT0).

### Histopathological margin infiltration by orientations

The histopathological workup of the main specimen (without re-excisions), showed an infiltration of 47 (4.5%) margins. The cranial and dorsal orientation most frequently showed margin involvement (14 positive margins, 1.3%).

### Margin assessment by CSR

In total, 1044 margins were analyzed with CSR (Table 2), of which 117 (11.2%) were radiologically positive. Nine (0.9%) were histologically and radiologically positive (true positive CSR). Based on the correct identification by CSR, these margins could be re-resected in the same surgery, potentially reaching a final negative margin state in the same surgery.

**Table 2:** Evaluation of Conventional Specimen Radiography on a margin level for the whole and for patients with clinical complete response versus no clinical complete response

	Overall cohort		Clinical complete response (cCR)		No clinical complete response (non-cCR)		Total––no. (%)
	CSR positive	CSR negative	CSR positive	CSR negative	CSR positive	CSR negative	–
Reference test positive*	9	38	1	9	8	29	–
Reference test negative**	108	889	51	449	57	440	–
Total––no. (%)	1044 (100%)		510 (100%)		534 (100%)		–
		(95% CI)		(95% CI)		(95% CI)	*p*-value***
Sensitivity––% (95% CI)	19.2%	(9.2–33.3%)	10.0%	(0.3–44.5%)	21.6%	(9.8–38.2%)	0.660
Specificity––% (95% CI)	89.2%	(87.1–91.0%)	89.8%	(86.8–92.3%)	88.5%	(85.4–91.2%)	0.542
PPV––% (95% CI)	7.7%	(3.6–14.1)	1.9%	(0.1–10.3%)	12.3%	(5.5–22.8%)	0.076
NPV––% (95% CI)	95.9%	(94.4–97.1%)	98.0%	(96.3–99.1%)	93.8%	(91.2–95.8)	0.001
Margin conversion through CSR––no. (%)	15	(1.4%)	1	(0.2%)	14	(2.6%)	
NNT		70		510	38		

*cCR* clinical complete response, *CSR* Conventional Specimen Radiography, *NACT* neoadjuvant chemotherapy, *NNT* number needed to treat, *NPV* negative predictive value, *PMR* positive margin rate, *PPV* positive predictive value

*Tumor infiltrated margin in histopathologic evaluation of the surgical specimen

**No tumor infiltrated margin in histopathologic evaluation of the surgical specimen

***For clinical complete response versus no clinical complete response

108 (10.3%) histopathologically clear margins were falsely assessed as positive by CSR. In these cases, healthy tissue was re-resected unnecessarily if the surgeon followed the recommendation for re-excision.

Of 927 radiologically negative margins, 38 (4.1%) were histologically infiltrated (false negative CSR). In these cases, no recommendation for re-excision was given based on CSR and the final margin status in the first surgery was positive (unless the surgeon performed a re-excision based on gross-inspection), resulting in the necessity for a second surgery.

### Comparison of margin assessment between cCR and non-cCR patients

Regarding all 1044 margins, CSR had a sensitivity of 19.2%, specificity of 89.2%, PPV of 7.7%, and NPV of 95.9%.

In the subgroup of cCR patients, the prevalence of histologically positive margins was 10 of 510 (2.0%). One of these margins was correctly diagnosed as radiologically positive (true positive CSR, 10.0%). In contrast, 51 of 500 histologically negative margins (10.2%) were false positive in CSR.

Compared to the non-cCR patients, there was no relevant difference in specificity (89.8% versus 88.5%, *p* = 0.542). Sensitivity (10.0% versus 21.6%, *p* = 0.660) and PPV (1.9% versus 12.3%, *p* = 0.076) were lower in the cCR subgroup, but the differences were not statistically significant.

### Intraoperative re-excisions and final positive margin status on case level

In 95 (54.6%) patients, at least one intraoperative re-excision was performed (Table 3). In 79 (83.2%) cases, this turned out to be unnecessary, because all margins were histopathologically negative. In 16 (9.2%) cases, margin infiltration was confirmed in histopathological examination. In 6 (3.4%) cases, all histologically infiltrated margins were correctly identified by CSR. In the remaining 10 (5.7%) cases, at least one histologically infiltrated margin was missed by CSR. Through intraoperative re-excisions, the number of infiltrated margins could be reduced from initially 47 (4.5%) to 32 (3.1%).

**Table 3:** It shows the effect of CSR-guided re-resections on the final margin status and reduction of secondary surgeries on case level

	Overall cohort	cCR after NACT	Non-cCR after NACT
Number of cases	174 (100%)	85 (100%)	89 (100%)
Initial PMR	25 (14.3%)	7 (8.2%)	18 (20.2%)
*True positive*	13 (7.5%)	3 (3.5%)	10 (11.2%)
*False positive*	62 (35.6%)	33 (38.8%)	29 (32.6%)
*True negative*	87 (50.0%)	45 (52.9%)	42 (47.2%)
*False negative*	12 (6.9%)	4 (4.7%)	8 (9.0%)
*Sensitivity*	52.0% (31.3–72.2%)	42.9% (9.9–81.6%)	55.6% (30.8–78.5%)
*Specificity*	58.4% (50.0–66.4%)	57.7% (46.0–68.8%)	59.2% (46.8–70.7%)
*PPV*	17.3% (9.6–27.8%)	8.3% (1.8–22.5%)	25.6% (13.0–42.1%)
*NPV*	87.9% (79.8–93.6%)	91.8% (80.4–97.7%)	84.0% (70.9–92.8%)
Final PMR	17 (9.8%)	6 (7.1%)	11 (12.4%)
Conversion of margin status through CSR	8 (4.6%)	1 (1.1%)	7 (7.9%)
NNT for conversion of margin status through CSR	22	85	13
Secondary surgeries	16 (9.2%)	6 (7.1%)	10 (11.2%)
Number of secondary surgeries avoided through CSR	7 (4%)	1 (1.1%)	6 (6.7%)
NNT to avoid secondary surgeries through CSR	25	85	15

*cCR* clinical complete response, *CSR* Conventional Specimen Radiography, *NACT* neoadjuvant chemotherapy, *NNT* number needed to treat, *PMR* positive margin rate in histology

### Detection of clip markers by CSR

154 of 158 (97.4%) clip markers used for preoperative localization were detected in CSR. In three of the six remaining cases, the marker was preoperatively found to be dislocated (by 10 mm, 10 mm, and 14 mm, respectively) and was therefore not directly targeted with the wire marking. In one case, the clip marker was detected neither in CSR nor in a postsurgical control mammography (therefore, the clip must have been removed during the surgery without noticing by the surgeon), and in one case it was identified macroscopically by the surgeon and removed before CSR. In 16 cases, no clip marker had been applied because microcalcifications were used for wire localization of the target lesion.

### Effect of CSR-guided resections on secondary procedures

In the whole cohort, 23 patients would have required further surgery if no margin assessment and no re-excisions had been carried out. Through intraoperative re-excisions based on CSR together with the gross assessment by the surgeon, clear margins were reached in 16 patients in the primary surgery. Thus, the rate of secondary procedures was reduced by 30.4%, resulting in a NNT of 25. In the cCR subgroup, the rate of secondary surgeries was reduced by 14.3% from seven to six patients by CSR-guided re-excisions. This translates to a NNT of 85 in the cCR subgroup.

### Comparison with the efficacy of CSR in a cohort of patients without NACT

In Table 4, we compare the results to the previously published data from a cohort without NACT from the same breast unit. On the margin level, we found a similar specificity (86.8%, versus 89.8%, *p* = 0.055), but a significantly lower sensitivity (19.2%) in the NACT cohort compared to the non-NACT cohort (36.8%; *p* = 0.012). The PPV is much lower after NACT than in the non-NACT cohort (7.7% vs. 25.6%; *p* =  < 0.001).

**Table 4:** Analysis of CSR in Patients with and without NACT on margin level

	No NACT^a^		NACT		*p*-value
Total margins	(*n* = 2826)	(95% CI)	(*n* = 1044)	(95% CI)	
Infiltrated margins	310 (11.0%)		47 (4.5%)		
Sensitivity	36.8%	(31.4–42.2%)	19.1%	(9.2–33.3%)	0.012*
Specificity	86.8%	(85.5–88.1%)	89.2%	(87.1–91.0%)	0.055*
Positive predictive value (PPV)	25.6%	(21.6–29.7%)	7.7%	(3.6–14.1%)	< 0.001*
Negative predictive value (NPV)	91.8%	(90.7–92.9%)	95.9%	(94.4–97.1%)	< 0.001*

*CI* confidence interval, *NACT* neoadjuvant chemotherapy

^**a**^data from a previously published analysis[11]

## Discussion

There are numerous studies on the use of CSR, but comparability of the results is limited, mostly because the accuracy of CSR is not evaluated on a margin level [12, 18–21]. A meta-analysis by Versteegden et al. reported a large range of sensitivity from 22 to 77%, specificity from 51 to 100%, and PPV from 51 to 100%, due to a large clinical and methodological diversity with low comparability of the studies [22]. While some studies [23–25], including a recent review by Gray et al. [26], indicate that CSR is not able to reduce the rate of positive margins and hence, the reoperation rate, Ciccarelli et al. and Chagpar et al. describe a reduction of the rate of second surgeries from 31 to 21% [12] and 37.8% to 28.9% [13].

The diagnostic accuracy of CSR in the present study including only patients after NACT was comparable to the results reported in the literature for non-selected cohorts, with sensitivity and specificity of 19.2% and 89.2%, respectively. However, the prevalence of initially positive margins was low in the overall cohort (*n* = 47, 4.5%) and even lower in the cCR cohort (*n* = 10, 2.0%). Consequently, only a few patients could potentially benefit from intraoperative re-excisions led by CSR. Accordingly, the NNT were 25 in all NACT patients and 85 in the cCR subgroup. This means that 84 of 85 patients with a cCR would not benefit from CSR, while one second surgery could be avoided. Whether this is an acceptable rate, has to be discussed from a patient-based, clinical perspective. Listening to our patients’ voice has gained more importance during the past years and should be considered as the tipping point in such controversial risk–benefit evaluations [28].

In cases with positive margin status, a secondary re-excision will be recommended to eliminate residual disease. Yet, some patients might decline a second surgery and thus do not achieve a finally negative margin. In this constellation, an intraoperative re-excision could have a relevant positive impact on oncologic safety.

Avoiding a second surgery also means a psychological advantage for the patients, and no second general anesthesia is needed. Lastly, esthetic outcome tends to be worse if two surgeries are necessary for definite treatment [29].

### False positives

The false positive rate of all assessed margins was 10.3%, which could lead to the unnecessary removal of healthy tissue if the recommendation for an intraoperative re-excision was followed. The impact of the re-excision on the esthetic outcome depends on the relation between the removed tissue and the breast size. [5, 30] The median specimen weight of the re-excisions in our study was 7.6 g (range 1; 41). In patients with small breasts, even minimal re-excisions with unnecessary removal of tissue could have a relevant effect on the esthetic outcome.

One explanation for the high false positive rate might be that in unclear cases the radiologist might intuitively tend to report a positive than a negative margin for maximum oncologic safety. In addition, there is no consensus on which radiological margin width should result in a recommendation for re-excision.

The shrinkage of the surgical specimen upon resection described as “pancake phenomenon” by Graham et al. results in apparently smaller safety margins and can contribute to a false positive margin assessment. Inadequate compression of the specimen during CSR can also lead to a distortion of the tumor-margin-relation [31].

Finally, a large part of the lesion can be necrotic or fibrotic due tumor regression after NACT, which can falsely appear like residual tumor [32, 33, 36].

### Comparison of cCR versus non-cCR cases

One would expect a very low prevalence of positive margins in patients with a cCR, since many of these will have no residual tumor. In our analysis, we found only 10/510 (2.0%) positive margins in the cCR cohort, compared to 37/534 (6.9%) in the non-cCR cases. As a result, the NNT of 85 to avoid one second surgery on case level is very high in the cCR group, versus 15 in the non-cCR group. In fact, when costs and risks of CSR-guided re-resections are balanced, CSR does not seem to be an appropriate tool for margin assessment in cCR patients. Apart from margin assessment, CSR is also used to test if the biopsy site clip was removed with the specimen as a real-time confirmation that surgery was performed in the correct localization of the tumor bed [27]. For this indication, CSR was very reliable in our analysis, since 154 or 97.4% of 158 clip markers were detected in CSR.

### Limitations

Since this is a retrospective study, no change of clinical practice can be recommended based on the results. Although we assessed 1044 margins, the power of the statistical analysis is limited by the low prevalence of positive margins especially in the cCR subgroup.

One limitation in the study design is that re-excisions could be performed not only based on the recommendation of CSR but also on the subjective assessment of the surgeons (e.g., according to their clinical impression after gross inspection of the specimen and palpation of the operation site). In future studies, the surgeon should be asked to document systematically what influenced their decision to perform a re-excision (recommendation from CSR versus subjective decision).

According to the current clinical standard in our unit, CSR was performed as standard digital mammography. Some authors argue that using tomosynthesis for margin assessment retrieves better results with a higher sensitivity [38, 39]. The potential benefit of tomosynthesis versus mammography is larger in patients with high breast density leading to a decreased diagnostic accuracy of CSR mammography.

An important source of error is the orientation and marking of the specimen. In the literature, rates of disorientation up to 31.1%, are reported, particularly in small specimens [40]. At our clinic, there is clear instruction for a standardized marking of the specimen orientation, which should help to reduce the error rate. Still, depending on size, form, and texture of the specimen, a clear marking can be challenging.

Due to the low prevalence of positive margins in the whole cohort, we did not perform a subgroup analysis by tumor biology. However, tumor subtypes differ regarding the patterns of tumor regression, which leads to heterogeneous radiological appearances. It seems likely that this also influences the accuracy of CSR. In future studies with sufficient sample size for subgroup analyses, tumor biological subtypes should be considered.

## Conclusion

The prevalence of initially positive margins after NACT and the sensitivity of CSR to detect them are low. A large proportion of patients might be overtreated by CSR-guided re-excisions. Balancing the benefit of a few spared second surgeries in relation to the much more frequent unnecessary or even harmful re-excisions after CSR, the use of CSR as a margin assessment tool cannot be generally recommended after NACT, particularly if a cCR was reached. Yet, CSR remains useful to document removal of the clip marker in the target lesion.

## Data Availability

Upon request from the corresponding author.

## Abbreviations

BCS: Breast conserving surgery
cCR: Clinical complete response
CI: Confidence interval
CSR: Conventional Specimen Radiography
NACT: Neoadjuvant chemotherapy
NNT: Number needed to treat
NPV: Negative predictive value
pCR: Pathological complete response
PMR: Positive margin rate
PPV: Positive predictive value

## References

[1] Fisher B, Anderson S, Bryant J, Margolese RG, Deutsch M, Fisher ER, Jeong JH, Wolmark N (2002). Twenty-year follow-up of a randomized trial comparing total mastectomy, lumpectomy, and lumpectomy plus irradiation for the treatment of invasive breast cancer. N Engl J Med.

[2] Veronesi U, Cascinelli N, Mariani L, Greco M, Saccozzi R, Luini A, Aguilar M, Marubini E (2002). Twenty-year follow-up of a randomized study comparing breast-conserving surgery with radical mastectomy for early breast cancer. N Engl J Med.

[3] Hofvind S, Holen A, Aas T, Roman M, Sebuodegard S, Akslen LA (2015). Women treated with breast conserving surgery do better than those with mastectomy independent of detection mode, prognostic and predictive tumor characteristics. European J Surg Oncol.

[4] Hwang ES, Lichtensztajn DY, Gomez SL, Fowble B, Clarke CA (2013). Survival after lumpectomy and mastectomy for early stage invasive breast cancer: the effect of age and hormone receptor status. Cancer.

[5] Hennigs A, Hartmann B, Rauch G, Golatta M, Tabatabai P, Domschke C, Schott S, Schutz F, Sohn C, Heil J (2015). Long-term objective esthetic outcome after breast-conserving therapy. Breast Cancer Res Treat.

[6] Foersterling E, Golatta M, Hennigs A, Schulz S, Rauch G, Schott S, Domschke C, Schuetz F, Sohn C, Heil J (2014). Predictors of early poor aesthetic outcome after breast-conserving surgery in patients with breast cancer: initial results of a prospective cohort study at a single institution. J Surg Oncol.

[7] Volders JH, Negenborn VL, Haloua MH, Krekel NMA, Jozwiak K, Meijer S, van den Tol PM (2018). Breast-specific factors determine cosmetic outcome and patient satisfaction after breast-conserving therapy: Results from the randomized COBALT study. J Surg Oncol.

[8] Heil J, Holl S, Golatta M, Rauch G, Rom J, Marme F, Gebauer G, Sohn C (2010). Aesthetic and functional results after breast conserving surgery as correlates of quality of life measured by a German version of the Breast Cancer Treatment Outcome Scale (BCTOS). Breast.

[9] Waljee JF, Hu ES, Ubel PA, Smith DM, Newman LA, Alderman AK (2008). Effect of esthetic outcome after breast-conserving surgery on psychosocial functioning and quality of life. J Clin Oncol.

[10] Horst KC, Smitt MC, Goffinet DR, Carlson RW (2005). Predictors of local recurrence after breast-conservation therapy. Clin Breast Cancer.

[11] Funk A, Heil J, Harcos A, Gomez C, Stieber A, Junkermann H, Hennigs A, Rauch G, Sinn HP, Riedel F (2020). Efficacy of intraoperative specimen radiography as margin assessment tool in breast conserving surgery. Breast Cancer Res Treat.

[12] Ciccarelli G, Di Virgilio MR, Menna S, Garretti L, Ala A, Giani R, Bussone R, Canavese G, Berardengo E (2007). Radiography of the surgical specimen in early stage breast lesions: diagnostic reliability in the analysis of the resection margins. Radiol Med.

[13] Chagpar AB, Butler M, Killelea BK, Horowitz NR, Stavris K, Lannin DR (2015). Does three-dimensional intraoperative specimen imaging reduce the need for re-excision in breast cancer patients? A prospective cohort study. Am J Surg.

[14] von Minckwitz G, Untch M, Blohmer JU, Costa SD, Eidtmann H, Fasching PA, Gerber B, Eiermann W, Hilfrich J, Huober J (2012). Definition and impact of pathologic complete response on prognosis after neoadjuvant chemotherapy in various intrinsic breast cancer subtypes. J Clin Oncol.

[15] Heil J, Kuerer HM, Pfob A, Rauch G, Sinn HP, Golatta M, Liefers GJ, Vrancken Peeters MJ (2020). Eliminating the breast cancer surgery paradigm after neoadjuvant systemic therapy: current evidence and future challenges. Ann Oncol.

[16] Interdisziplinäre S3-Leitlinie für die Diagnostik, Therapie und Nachsorge des Mammakarzinoms [http://www.awmf.org/uploads/tx_szleitlinien/032-045OL_k_S3__Brustkrebs_Mammakarzinom_Diagnostik_Therapie_Nachsorge_2012-07.pdf].

[17] Fachgesellschaften AdWM: Interdisziplinäre S3-Leitlinie für die Früherkennung, Diagnostik, Therapie und Nachsorge des Mammakarzinoms. 2017.

[18] McCormick JT, Keleher AJ, Tikhomirov VB, Budway RJ, Caushaj PF (2004). Analysis of the use of specimen mammography in breast conservation therapy. Am J Surg.

[19] Hisada T, Sawaki M, Ishiguro J, Adachi Y, Kotani H, Yoshimura A, Hattori M, Yatabe Y, Iwata H (2016). Impact of intraoperative specimen mammography on margins in breast-conserving surgery. Mol Clin Oncol.

[20] Schmachtenberg C, Engelken F, Fischer T, Bick U, Poellinger A, Fallenberg EM (2012). Intraoperative specimen radiography in patients with nonpalpable malignant breast lesions. Rofo.

[21] Mazouni C, Rouzier R, Balleyguier C, Sideris L, Rochard F, Delaloge S, Marsiglia H, Mathieu MC, Spielman M, Garbay JR (2006). Specimen radiography as predictor of resection margin status in non-palpable breast lesions. Clin Radiol.

[22] Versteegden DPA, Keizer LGG, Schlooz-Vries MS, Duijm LEM, Wauters CAP, Strobbe LJA (2017). Performance characteristics of specimen radiography for margin assessment for ductal carcinoma in situ: a systematic review. Breast Cancer Res Treat.

[23] Rua C, Lebas P, Michenet P, Ouldamer L (2012). Evaluation of lumpectomy surgical specimen radiographs in subclinical, in situ and invasive breast cancer, and factors predicting positive margins. Diagn Interv Imaging.

[24] Laws A, Brar MS, Bouchard-Fortier A, Leong B, Quan ML (2018). Does intra-operative margin assessment improve margin status and re-excision rates? A population-based analysis of outcomes in breast-conserving surgery for ductal carcinoma in situ. J Surg Oncol.

[25] Laws A, Brar MS, Bouchard-Fortier A, Leong B, Quan ML (2016). Intraoperative margin assessment in wire-localized breast-conserving surgery for invasive cancer: a population-level comparison of techniques. Ann Surg Oncol.

[26] Gray RJ, Pockaj BA, Garvey E, Blair S (2017). Intraoperative margin management in breast-conserving surgery: a systematic review of the literature. Ann Surg Oncol.

[27] Green RT, Weiser R, Golan O, Menes TS (2021). In search of the lost clip: outcome of women after needle-guided lumpectomy of a marking clip. Ann Surg Oncol.

[28] Heil J, Pfob A (2020). Patients should be the tipping point of individualizing breast cancer surgery: Commentary on 'Eliminating the breast cancer surgery paradigm after neoadjuvant systemic therapy: current evidence and future challenges'. Ann Oncol.

[29] Dahlback C, Manjer J, Rehn M, Ringberg A (2016). Determinants for patient satisfaction regarding aesthetic outcome and skin sensitivity after breast-conserving surgery. World J Surg Oncol.

[30] Cochrane RA, Valasiadou P, Wilson AR, Al-Ghazal SK, Macmillan RD (2003). Cosmesis and satisfaction after breast-conserving surgery correlates with the percentage of breast volume excised. Br J Surg.

[31] Clingan R, Griffin M, Phillips J, Coberly W, Jennings W (2003). Potential margin distortion in breast tissue by specimen mammography. Arch Surg.

[32] Helvie MA, Joynt LK, Cody RL, Pierce LJ, Adler DD, Merajver SD (1996). Locally advanced breast carcinoma: accuracy of mammography versus clinical examination in the prediction of residual disease after chemotherapy. Radiology.

[33] Moskovic EC, Mansi JL, King DM, Murch CR, Smith IE (1993). Mammography in the assessment of response to medical treatment of large primary breast cancer. Clin Radiol.

[34] Goorts B, van Nijnatten TJ, de Munck L, Moossdorff M, Heuts EM, de Boer M, Lobbes MB, Smidt ML (2017). Clinical tumor stage is the most important predictor of pathological complete response rate after neoadjuvant chemotherapy in breast cancer patients. Breast Cancer Res Treat.

[35] Kagihara JA, Andress M, Diamond JR (2020). Nab-paclitaxel and atezolizumab for the treatment of PD-L1-positive, metastatic triple-negative breast cancer: review and future directions. Expert Rev Precis Med Drug Dev.

[36] Schaefgen B, Mati M, Sinn HP, Golatta M, Stieber A, Rauch G, Hennigs A, Richter H, Domschke C, Schuetz F (2016). Can routine imaging after neoadjuvant chemotherapy in breast cancer predict pathologic complete response?. Ann Surg Oncol.

[37] Pfob A, Sidey-Gibbons C, Lee HB, Tasoulis MK, Koelbel V, Golatta M, Rauch GM, Smith BD, Valero V, Han W (2021). Identification of breast cancer patients with pathologic complete response in the breast after neoadjuvant systemic treatment by an intelligent vacuum-assisted biopsy. Eur J Cancer.

[38] Amer HA, Schmitzberger F, Ingold-Heppner B, Kussmaul J, El Tohamy MF, Tantawy HI, Hamm B, Makowski M, Fallenberg EM (2017). Digital breast tomosynthesis versus full-field digital mammography-Which modality provides more accurate prediction of margin status in specimen radiography?. Eur J Radiol.

[39] Park KU, Kuerer HM, Rauch GM, Leung JWT, Sahin AA, Wei W, Li Y, Black DM (2019). Digital breast tomosynthesis for intraoperative margin assessment during breast-conserving surgery. Ann Surg Oncol.

[40] Molina MA, Snell S, Franceschi D, Jorda M, Gomez C, Moffat FL, Powell J, Avisar E (2009). Breast specimen orientation. Ann Surg Oncol.

